# Supplementary material for: Effect of Temperature and Radiation on Indica Rice Yield and Quality in Middle Rice Cropping System
Source: Plants (Basel). 2022 Oct 13;11(20):2697. doi: 10.3390/plants11202697 (PMC9607267; doi:10.3390/plants11202697)
Supplement: Supplementary file 1 [file plants-11-02697-s001.zip › plants-1938161-supplementary.pdf]

Different ecological regions including Yuanyang in Henan province (35.3° N, 114.1° E), Tongcheng in Anhui province (31.0° N, 116.9° E), Fuyang in Zhejiang province (30.0° N, 119.9° E), Yifeng in Jiangxi province (28.4° N, 114.8° E).

Table S1. The priperties of soil in different cultivated areas in the present study.

Area	pH	organic matter(g kg^{-1})	total N (g kg^{-1})	available Olsen-P (mg kg^{-1})	exchangeable K (mg kg^{-1})
**Yuanyang**	5.9	15.6	1.7	11.1	109.8
**Tongcheng**	6.0	23.6	1.4	16.2	94.3
**Fuyang**	6.8	28.1	2.1	26.5	68.2
**Yifeng**	5.9	31.3	2.5	11.7	66.3

Table S2 the growth period of six cultivars among different cultivated areas in two years

Variety	Area	2019 Sowing date	2019 Heading date	2019 Maturity date	2020 Sowing date	2020 Heading date	2020 Maturity date
**Taoyouxiangzhan**	Yuanyang	16-May	20-Aug	27-Sep	16-May	22-Aug	24-Sep
	Tongchen	10-May	23-Aug	6-Oct	10-May	22-Aug	7-Oct
	Fuyang	17-May	20-Aug	20-Sep	17-May	18-Aug	19-Sep
	Yifeng	22-May	11-Aug	13-Sep	22-May	9-Aug	11-Sep
**Taiyou398**	Yuanyang	16-May	7-Aug	20-Sep	16-May	10-Aug	22-Sep
	Tongchen	10-May	6-Aug	21-Sep	10-May	5-Aug	20-Sep
	Fuyang	17-May	13-Aug	10-Sep	17-May	12-Aug	8-Sep
	Yifeng	22-May	1-Aug	29-Aug	22-May	2-Aug	30-Aug
**Fengliangyou4hao**	Yuanyang	16-May	25-Aug	28-Sep	16-May	22-Aug	28-Sep
	Tongchen	10-May	28-Aug	10-Oct	10-May	29-Aug	8-Oct
	Fuyang	17-May	24-Aug	23-Sep	17-May	22-Aug	22-Sep
	Yifeng	22-May	19-Aug	24-Sep	22-May	18-Aug	25-Sep
**Jingliangyouhuazhan**	Yuanyang	16-May	25-Aug	28-Sep	16-May	22-Aug	28-Sep
	Tongchen	10-May	27-Aug	9-Oct	10-May	25-Aug	7-Oct
	Fuyang	17-May	24-Aug	23-Sep	17-May	22-Aug	24-Sep
	Yifeng	22-May	21-Aug	26-Sep	22-May	20-Aug	27-Sep
**Wandao153**	Yuanyang	16-May	25-Aug	28-Sep	16-May	22-Aug	28-Sep
	Tongchen	10-May	25-Aug	4-Oct	10-May	23-Aug	6-Oct
	Fuyang	17-May	24-Aug	23-Sep	17-May	22-Aug	20-Sep
	Yifeng	22-May	18-Aug	23-Sep	22-May	17-Aug	21-Sep
**IIyou838**	Yuanyang	16-May	25-Aug	1-Oct	16-May	22-Aug	28-Sep
	Tongchen	10-May	25-Aug	1-Oct	10-May	23-Aug	1-Oct
	Fuyang	17-May	20-Aug	20-Sep	17-May	21-Aug	21-Sep
	Yifeng	22-May	19-Aug	22-Sep	22-May	18-Aug	20-Sep

Table S3 grain yield and quality of each cultivar across four areas and two years

Year	Variety	Area	Grain yield (t hm^{-2})	Head rice rate (%)	Chalky grain rate(%)	Chalkiness level(%)
2019	Taoyouxiangzhan	Yuanyang	8.42 ± 0.16	71.44 ± 1.11	5.99 ± 0.23	1.24 ± 0.11
		Tongchen	7.92 ± 0.31	70.3 ± 0.98	6.15 ± 0.12	1.75 ± 0.09
		Fuyang	8.32 ± 0.27	70.0 ± 0.64	3.49 ± 0.21	0.92 ± 0.06
		Yifeng	4.77 ± 0.21	68.7 ± 1.02	6.50 ± 0.35	1.62 ± 0.07
2020	Taoyouxiangzhan	Yuanyang	7.39 ± 0.29	70.8 ± 0.89	4.73 ± 0.31	1.98 ± 0.11
		Tongchen	8.53 ± 0.41	69.7 ± 0.45	4.10 ± 0.27	0.99 ± 0.05
		Fuyang	10.66 ± 0.66	70.4 ± 0.67	11.92 ± 0.81	2.70 ± 0.12
		Yifeng	6.33 ± 0.22	70.9 ± 0.95	3.98 ± 0.20	0.94 ± 0.06
2019	Tanyou398	Yuanyang	9.98 ± 0.49	70.1 ± 1.01	5.20 ± 0.41	1.32 ± 0.09
		Tongchen	8.45 ± 0.27	70.0 ± 0.49	7.60 ± 0.50	1.96 ± 0.07
		Fuyang	7.51 ± 0.38	68.2 ± 0.59	7.00 ± 0.39	1.75 ± 0.12
		Yifeng	5.35 ± 0.21	67.1 ± 0.68	12.00 ± 0.31	3.58 ± 0.21
2020	Tanyou398	Yuanyang	7.60 ± 0.35	70.3 ± 0.79	12.00 ± 0.47	3.05 ± 0.15
		Tongchen	5.05 ± 0.23	67.6 ± 0.63	9.10 ± 0.56	2.76 ± 0.17
		Fuyang	8.35 ± 0.19	64.7 ± 0.63	16.40 ± 0.92	4.73 ± 0.23
		Yifeng	7.05 ± 0.26	67.7 ± 0.96	11.30 ± 0.67	3.84 ± 0.21
2019	Fengliangyou4hao	Yuanyang	10.1 ± 0.56	69.7 ± 0.65	6.30 ± 0.55	1.51 ± 0.09
		Tongchen	8.68 ± 0.32	70.2 ± 0.97	14.20 ± 0.46	3.99 ± 0.32
		Fuyang	8.91 ± 0.24	72.0 ± 1.04	4.50 ± 0.21	1.04 ± 0.08
		Yifeng	6.20 ± 0.19	68.0 ± 0.96	13.40 ± 0.41	3.89 ± 0.12
2020	Fengliangyou4hao	Yuanyang	8.63 ± 0.27	71.0 ± 0.51	15.20 ± 0.86	4.59 ± 0.13
		Tongchen	9.70 ± 0.35	72.1 ± 0.69	21.10 ± 1.01	4.83 ± 0.22
		Fuyang	10.61 ± 0.69	66.9 ± 2.01	22.30 ± 0.97	6.36 ± 0.31
		Yifeng	9.07 ± 0.39	69.2 ± 1.03	14.90 ± 0.85	4.51 ± 0.11
2019	Jingliangyouhuazhan	Yuanyang	9.57 ± 0.41	70.2 ± 0.98	15.83 ± 0.76	3.45 ± 0.13
		Tongchen	8.05 ± 0.36	70.8 ± 0.63	7.00 ± 0.56	2.25 ± 0.07
		Fuyang	7.59 ± 0.28	70.7 ± 0.76	8.10 ± 0.46	1.70 ± 0.09
		Yifeng	6.98 ± 0.43	68.0 ± 0.35	20.20 ± 0.46	4.97 ± 0.17
2020	Jingliangyouhuazhan	Yuanyang	8.15 ± 0.57	70.1 ± 0.49	8.00 ± 0.57	3.29 ± 0.11

Continue

	Variety	Area	Grain yield ($t\ hm^{-2}$)	Head rice rate (%)	Chalky grain rate(%)	Chalkiness level(%)
		Tongchen	11.78 ± 0.75	70.9 ± 0.71	6.30 ± 0.45	1.56 ± 0.09
		Fuyang	11.1 ± 0.64	69.0 ± 0.89	10.21 ± 0.59	2.84 ± 0.11
		Yifeng	8.02 ± 0.29	71.2 ± 1.50	7.12 ± 0.65	2.05 ± 0.09
	Wandao153	Yuanyang	9.72 ± 0.33	70.2 ± 1.23	11.28 ± 0.66	2.56 ± 0.07
2019		Tongchen	7.63 ± 0.43	69.8 ± 1.51	16.39 ± 0.79	4.65 ± 0.25
		Fuyang	9.30 ± 0.56	73.0 ± 1.01	12.89 ± 0.96	3.19 ± 0.13
		Yifeng	6.84 ± 0.26	67.7 ± 0.99	25.81 ± 2.01	9.00 ± 0.25
	Wandao153	Yuanyang	8.33 ± 0.61	71.2 ± 0.87	17.92 ± 0.49	5.99 ± 0.12
2020		Tongchen	12.38 ± 0.89	72.6 ± 0.68	13.61 ± 0.89	3.14 ± 0.15
		Fuyang	11.04 ± 0.47	69.8 ± 1.11	30.22 ± 1.23	8.22 ± 0.21
		Yifeng	8.75 ± 0.67	70.9 ± 1.03	26.73 ± 0.75	9.40 ± 0.27
	IIyou838	Yuanyang	9.30 ± 0.77	70.7 ± 1.07	34.14 ± 1.56	9.12 ± 0.25
2019		Tongchen	7.77 ± 0.69	69.8 ± 0.95	9.61 ± 0.68	2.76 ± 0.14
		Fuyang	7.87 ± 0.45	69.6 ± 0.93	40.70 ± 2.13	12.72 ± 0.36
		Yifeng	7.69 ± 0.35	65.9 ± 0.76	59.11 ± 1.96	19.53 ± 0.53
	IIyou838	Yuanyang	9.08 ± 0.29	67.7 ± 0.89	25.63 ± 0.75	10.19 ± 0.57
2020		Tongchen	10.22 ± 0.68	72.0 ± 1.01	10.84 ± 0.68	2.48 ± 0.11
		Fuyang	10.40±0.99	70.5 ± 1.03	39.91 ± 0.95	10.52 ± 0.48
		Yifeng	9.14±0.75	67.2 ± 0.89	35.10 ± 1.79	11.42 ± 0.37

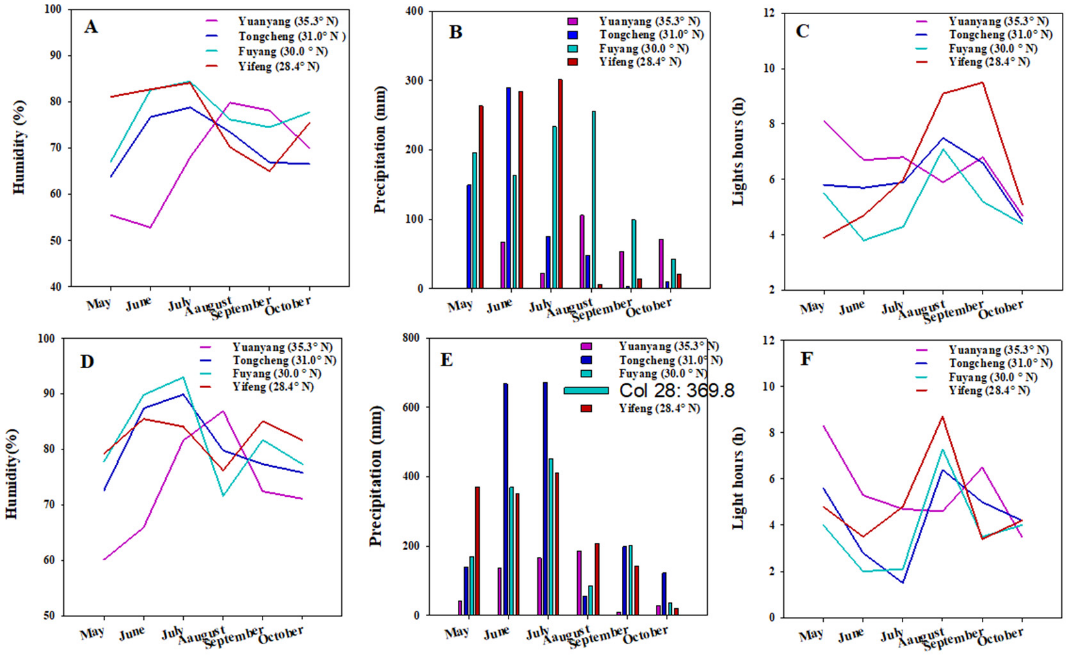

Figure.S1. The humidity, precipitation and light hours conditions during rice growth seasons across four cultivated areas and two years. A, B and C are for 2019; D, E and F are for 2020.
